# Iron Replacement and Redox Balance in Non-Anemic and Mildly Anemic Iron Deficiency COPD Patients: Insights from a Clinical Trial

**DOI:** 10.3390/biomedicines9091191

**Published:** 2021-09-10

**Authors:** Maria Pérez-Peiró, Clara Martín-Ontiyuelo, Anna Rodó-Pi, Lucilla Piccari, Mireia Admetlló, Xavier Durán, Diego A. Rodríguez-Chiaradía, Esther Barreiro

**Affiliations:** 1 Pulmonology Department-Muscle Wasting and Cachexia in Chronic Respiratory Diseases and Lung Cancer Research Group, IMIM-Hospital del Mar, Parc de Salut Mar, Health and Experimental Sciences Department (CEXS), Universitat Pompeu Fabra (UPF), Parc de Recerca Biomèdica de Barcelona (PRBB), 08003 Barcelona, Spain; mperez4@imim.es (M.P.-P.); CMartinOntiyuelo@psmar.cat (C.M.-O.); arodo@psmar.cat (A.R.-P.); lucilla.piccari@gmail.com (L.P.); madmetllo@psmar.cat (M.A.); darodriguez@parcdesalutmar.cat (D.A.R.-C.); 2 Centro de Investigación en Red de Enfermedades Respiratorias (CIBERES), Instituto de Salud Carlos III (ISCIII), 08003 Barcelona, Spain; 3 Scientific and Technical Department, Hospital del Mar-IMIM, 08003 Barcelona, Spain; xduran@imim.es

**Keywords:** non-anemic iron deficient (NAID) COPD patients, pathophysiological mechanisms, iron metabolism, redox balance, iron replacement as a therapeutic opportunity

## Abstract

In COPD patients, non-anemic iron deficiency (NAID) is a common systemic manifestation. We hypothesized that in COPD patients with NAID, iron therapy may improve systemic oxidative stress. The FACE (Ferinject assessment in patients with COPD and iron deficiency to improve exercise tolerance) study was a single-blind, unicentric, parallel-group, placebo-controlled clinical trial (trial registry: 2016-001238-89). Sixty-six patients were enrolled (randomization 2:1): iron arm, *n* = 44 and placebo arm, *n* = 22, with similar clinical characteristics. Serum levels of 3-nitrotyrosine, MDA-protein adducts, and reactive carbonyls, catalase, superoxide dismutase (SOD), glutathione, Trolox equivalent antioxidant capacity (TEAC), and iron metabolism biomarkers were quantified in both groups. In the iron-treated patients compared to placebo, MDA-protein adducts and 3-nitrotyrosine serum levels significantly declined, while those of GSH increased and iron metabolism parameters significantly improved. Hepcidin was associated with iron status parameters. This randomized clinical trial evidenced that iron replacement elicited a decline in serum oxidative stress markers along with an improvement in GSH levels in patients with stable severe COPD. Hepcidin may be a surrogate biomarker of iron status and metabolism in patients with chronic respiratory diseases. These findings have potential clinical implications in the management of patients with severe COPD.

## 1. Introduction

Chronic obstructive pulmonary disease (COPD) patients frequently experience acute exacerbations that further deteriorate their lung function, as well as other systemic manifestations, which are very common in these patients, leading to poor exercise capacity and quality of life [1,2,3]. The homeostasis of iron is a complex process tightly regulated by many chemical reactions and molecules. Oxygen transport, mitochondrial respiration, DNA synthesis, and cellular metabolism, are processes in which iron plays a crucial role [4,5]. Lung function, as well as other systemic manifestations, are very common in these patients [6]. In these patients, anemia and NAID were of prognosis value in clinical settings during both acute exacerbations and in the stable disease [7,8,9,10]. Moreover, alterations in iron metabolism also take place in other conditions during acute and chronic phases, particularly in hyperinflammatory states [11,12]. Hepcidin, a hormone involved in the regulation of iron flow between plasma and reservoirs and from the gastrointestinal tract, is also a marker of inflammation as an acute phase reactant [13,14,15] and can be regulated by many other factors [16,17,18,19].

Oxidative stress, defined as the imbalance between oxidants and antioxidants, underlies the pathophysiology of acute and chronic lung diseases, including COPD [20,21,22]. Systemic manifestations are also characterized by a rise in oxidative stress events both at the systemic level and in specific organs, such as skeletal muscles in patients with severe COPD [1,23,24]. Several cellular and non-cellular processes, such as the Fenton reaction, characterized by the interaction between hydrogen peroxide and iron, contribute to the synthesis of oxidants within the cells [25,26,27].

Indirect indices of oxidative stress are commonly used to assess the oxidative status in tissues and fluids under different conditions [28,29,30,31]. As such, 3-nitrotyrosine, malondialdehyde (MDA)-protein adducts and reactive carbonyls are counted among the most frequently detected markers of oxidative stress [21,31,32]. Enzymatic and non-enzymatic antioxidant molecules scavenge oxidant species in tissues in order to keep their levels within the normal ranges [33,34]. Indirect markers of antioxidants are also commonly explored in tissues and cells [27,28]. Oxidants that escape the action of antioxidants modify key cellular structures that favor an oxidative environment. 

In a recent study [35], treatment with iron for four weeks induced a significant improvement in clinical outcomes, including the endurance time of the maximal exercise capacity. Iron replacement for several weeks also elicited beneficial effects in patients with ID anemia, particularly in oxidative stress markers, characterized by a decline after the treatment compared to baseline [36,37]. Whether levels of indirect oxidative stress markers may also decline in patients with severe COPD following a protocol of iron replacement remains to be answered. Likewise, whether levels of antioxidant markers may increase in response to iron treatment still needs to be confirmed. 

On this basis, we hypothesized that in COPD patients with NAID, treatment with iron might improve systemic oxidative stress and antioxidant levels. Specifically, redox balance as measured by the identification of prooxidant and antioxidant markers was thoroughly assessed in the systemic compartment of the study patients. Hence, a clinical trial was specifically designed to address this question. Accordingly, the study objectives were to analyze the following markers in placebo and iron-treated groups of patients: (1) serum levels of 3-nitrotyrosine, MDA-protein adducts, and reactive carbonyls, (2) antioxidants, such as catalase, superoxide dismutase (SOD), glutathione, and Trolox equivalent antioxidant capacity (TEAC), and (3) iron metabolism, including hepcidin. 

## 2. Materials and Methods

### 2.1. Study Population, Design, and Ethics

**Figure 1:**
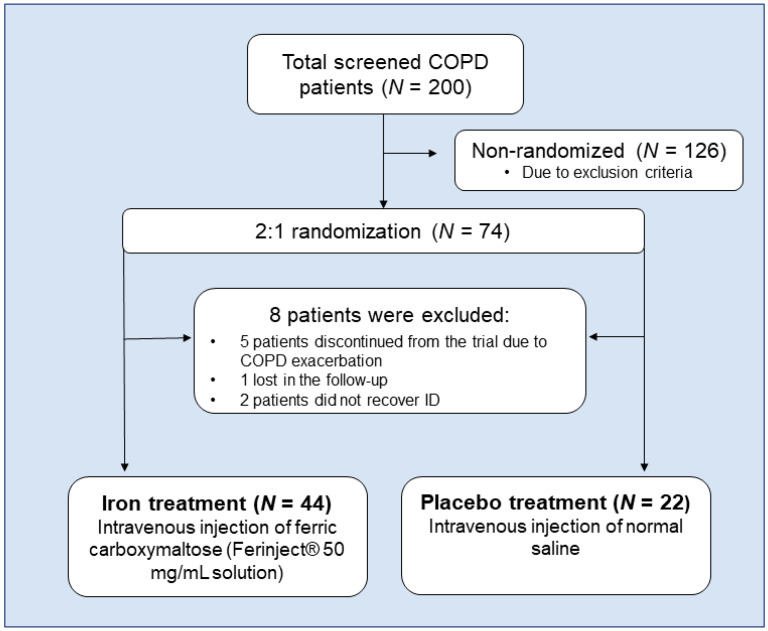
Flowchart of the FACE trial according to CONSORT guidelines. Definition of abbreviations: COPD, chronic obstructive pulmonary disease; ID, iron deficiency.

The FACE (Ferinject assessment in patients with COPD and iron deficiency to improve exercise tolerance) study was a single-blind, unicentric, parallel-group, placebo-controlled clinical trial following the Consolidated Standards of Reporting Trials (CONSORT) guidelines [38]. FACE was registered in EudraCT with the number 2016-001238-89. As seen in Figure 1, a total of 200 patients were consecutively screened to participate in this trial from the COPD Clinics in the Department of Respiratory Medicine at Hospital del Mar (Barcelona, Spain) from January 2018 to January 2020. The research followed the guidelines of the World Medical Association for Research in Humans (Seventh revision of the Declaration of Helsinki, Fortaleza, Brazil, 2013). The study was approved by the local Ethics Committee at Hospital del Mar (CEIm Parc de Salut Mar, registration # 2016/6730). All the participating patients signed their written informed consent. For more detailed information on the clinical and physiological results, see reference [35].

### 2.2. Inclusion Criteria

Eligible participants were Chronic obstructive pulmonary disease (COPD) patients who were diagnosed according to the Global Strategy of Management of COPD patients (GOLD) criteria [39] and with NAID (hemoglobin > 12 g/dL in women and >13 g/dL in men, ferritin < 100 ng/mL or ferritin 100–299 ng/mL with a transferrin saturation < 20%) [40,41] or with mild anemia (hemoglobin levels between 12 and 13 g/dL in men, and 11 and 12 g/dL in women) [42]. From the initial 200 patients, only 37% (*N* = 74) fulfilled the required criteria to participate in this trial: clinically stable for at least 8 weeks prior to study entry, and the age range was from 40 to 80 years old. During the course of the study, five patients were excluded due to a COPD exacerbation, one was lost in the follow-up, and two did not respond to iron replacement. Sixty-six patients were finally enrolled and were subsequently randomized to intravenous iron (*N* = 44) or placebo (*N* = 22) treatment in a 2:1 ratio (Figure 1). Out of the 44 patients in the treated group, four (9.1%) presented mild anemia, while in the placebo group, only one patient exhibited mild anemia.

### 2.3. Iron Replacement: Placebo and Treatment Arms

In the iron-treated patients, ferric carboxymaltose solution (Ferinject^®^, Vifor, St. Gallen, Switzerland), 10 mL-injections containing either 500 mg ferric carboxymaltose (diluted in 250 mL 0.9% normal saline) or 20 mL, depending on the patient’s body weight and hemoglobin levels following the manufacturer’s product label of the Spanish Agency of Drugs, were administered for 15 min while the patient was resting. In the placebo patients, 250 mL 0.9% normal saline was injected for 15 min while the patients were also resting (Figure 1). In order to keep the blinding process to the patients, the clinical trial pharmacist prepared both placebo and ferric carboxymaltose infusions using an opaque dark coverage to avoid the potential identification by the patients of the treatment arm.

### 2.4. Exclusion Criteria

Exclusion criteria for study patients included: (1) Cardiovascular (heart failure with left ventricle fraction ejection below 60%), neurological, kidney, musculoskeletal alterations, or uncontrolled psychiatric disorders; (2) other respiratory diseases (e.g., asthma, bronchiectasis, long-term oxygen therapy, or obstructive sleep apnea syndrome); (3) obesity (body mass index > 30 kg/m^2^); (4) history of potentially bleeding conditions, pregnancy or breast-feeding, chronic liver disease, or active oncologic disease; (5) allergy or hypersensitivity to parenteral iron administration or any of the excipients; (6) polycythemia, hemoglobin ≤ 12 g/dL in men and ≤11 g/dL in women; (7) treatment in the previous month with erythropoietin, iron (oral or intravenous), transfusions, or any kind of sex-related hormones; (8) antibiotics intake and/or systemic corticosteroids in the previous three months, and (9) participation in another clinical trial simultaneously or in the previous year.

### 2.5. Anthropometric and Lung Function Assessment

Body mass index (BMI) and determination of fat-free mass index (FFMI) were evaluated using bioelectrical impedance [43,44]. Evaluation of lung function was determined through spirometry, static lung volumes, diffusion capacity, and blood gases using well-established procedures and reference values [45,46,47].

### 2.6. Blood Samples

Blood samples were obtained from the arm vein after an overnight fasting period at baseline (before treatment administration) and four weeks after intravenous ferric carboxymaltose or the placebo administration. The following blood parameters: hemoglobin, hematocrit, mean corpuscular (erythrocyte) volume (MCV), mean corpuscular hemoglobin (MCH), mean corpuscular hemoglobin concentration (MCHC), serum iron, transferrin, transferrin saturation, serum ferritin, and soluble transferrin receptor were evaluated. In order to determine levels of the target redox balance markers, blood samples were collected into vacuette^®^ serum tubes (with clot activator). Blood samples were centrifuged at 1600× *g* for 15 min to obtain the serum, which was preserved for the study purposes. All the patient samples were immediately stored at −80 °C for further use.

### 2.7. Molecular Biology Analysis

Interleukin 6 (IL-6). Protein levels of IL-6 were quantified in serum from all of the study subjects using a RayBio^®^ Human IL-6 ELISA kit (RayBiotech, Norcross, GA, USA) following the manufacturer’s instructions and previously described methodologies [30]. Once samples and reagents were equilibrated to room temperature, 100 µL of serum and standards were added to each well. The plate was incubated at room temperature for 2.5 h. The samples were washed four consecutive times to be incubated at room temperature with the biotinylated antibody for one hour. After four more washes, streptavidin solution was added to each well and samples were incubated at room temperature for 45 additional minutes. Subsequently, after four washes, TMB substrate reagent was poured into each well at room temperature in the dark for 30 min. Finally, the enzymatic reaction was stopped upon the addition of the stop reaction. The absorbance was read at 450 nm in all the samples. A standard curve was always generated with each assay run. Intra-assay coefficients of variation for all the samples ranged from 0.12% to 9.56%.

Hepcidin-25. The determination of Hepcidin-25 concentration in serum samples was analyzed using a Human hepcidin (Hepc) ELISA kit (Biorbyt, Cambridgeshire, UK) following the manufacturer’s instructions. All reagents and samples needed to be equilibrated to room temperature before being used. Briefly, 50 µL 5-fold diluted samples or standard and 50 µL HRP-conjugate were added in each well of the hepcidin antibody pre-coated microplate. Samples were incubated at 37 °C for one hour and were then washed 3 more times. Subsequently, 50 µL substrate A and 50 µL substrate B were poured and incubated at 37 °C for 15 min. Finally, the enzymatic reaction was terminated by adding 50 µL of the stop solution. Immediately afterward, the optical densities (absorbance) in each well were determined at 450 nm wavelength. A standard curve was always generated with each assay run. Intra-assay coefficients of variation for all the samples ranged from 0.10% to 10.57%. No reference values for serum hepcidin concentration in patients have been reported so far. Nonetheless, similar values to the ones shown in the current investigation were previously published [48].

3-Nitrotyrosine (3-NT). The determination of 3-nitrotyrosine concentration in serum samples was analyzed using an Elabscience^®^ 3-NT(3-Nytrotyrosine) ELISA kit (Elabscience, Houston, TX, USA) following the manufacturer’s instructions and previously described methodologies [49]. All reagents needed to be equilibrated at room temperature before the beginning of the ELISA procedures. Thus, 50 µL 7-fold diluted serum samples and standards were added in the corresponding wells of the 3-nitrotyrosine antibody pre-coated microplates. Subsequently, samples were incubated at 37 °C with the biotinylated antibody for 45 min. After three consecutive washes, HRP conjugate working solution was added to each well, and the samples were then incubated at 37 °C for 30 min. Finally, after five additional washes, the samples were incubated at 37 °C with the substrate reagent for 15 more minutes. Following this incubation, the enzyme–substrate reaction was stopped by adding the stop solution. The optical densities in each well were determined by reading the absorbance of the samples at 450 nm wavelength. A standard curve was always generated with each assay run. Intra-assay coefficients of variation for all the samples ranged from 0.02% to 9.48%.

Malondialdehyde-protein adducts. Levels of MDA-protein adducts were measured in serum using the OxiSelect^TM^ MDA Adduct Competitive ELISA Kit (Cell Biolabs, Inc., San Diego, CA, USA) following the specific manufacturer’s instructions and previously described methodologies [29]. In brief, 50 µL serum and MDA-BSA standards were added to the MDA conjugate preabsorbed ELISA plate. The samples were then incubated at room temperature for 10 min. Subsequently, the primary antibody was added and incubated at room temperature for one hour. After three washes, the samples were incubated with the HRP conjugated secondary antibody at room temperature for an hour. Subsequently, the plate was washed three more times, and the substrate solution was incubated at room temperature for 20 min. Afterward, the enzyme reaction was stopped by adding 100 µL of the stop solution to each well. The absorbances were read in each well at 450 nm wavelength. A standard curve was always generated with each assay run. Intra-assay coefficients of variation for all the samples ranged from 0.02% to 9.38%. 

Protein carbonylation. Protein carbonyls levels in the serum samples were determined using an OxiSelect^TM^ Protein Carbonyl ELISA kit (Cell Biolabs, Inc., San Diego, CA, USA) following the manufacturer’s instructions and previously described methodologies [29,49]. The protein concentrations of serum samples were determined using Bradford methodologies. Briefly, 10 µg/mL protein sample and BSA standards were added to the corresponding wells. The samples were incubated at 37 °C for two hours. Following three washes, dinitrophenylhydrazine (DNPH) was added to each well, and the samples were incubated at room temperature in the dark for 45 min. After seven washes, the samples were incubated with blocking solution for one hour and were also washed three more times. Then, the primary anti-DNP antibody was poured into each well, and the samples were then incubated at room temperature for one hour. Micro-plate wells were washed three additional times to let the samples incubate with HRP conjugated secondary antibody at room temperature for one hour. Finally, after five more washes, the samples were incubated with substrate solution for 15 min. The enzymatic reaction was terminated by adding the stop solution to each well. Absorbance was read at 450 nm. A standard curve was always generated with each assay run. Intra-assay coefficients of variation for all the samples ranged from 0.02% to 8.16%.

Reduced glutathione (GSH). GSH was measured in the serum samples using the Human Reduced Glutathione (GSH) ELISA Kit (MyBioSource, San Diego, CA, USA) following the specific manufacturer’s instructions and previously described methodologies [49]. All reagents and samples needed to be equilibrated to room temperature (18–25 °C) before starting the procedure. Briefly, 50 µL samples and standards were added to each well and incubated with horseradish (HRP)-conjugate reagent at 37 °C for 60 min. After four washes, 50 µL chromogen solution A and 50 µL chromogen solution B were added to each well. The samples were then incubated at 37 °C in the dark for 15 min. Finally, 50 µL of the stop solution was incubated for five minutes, and the absorbance in each sample was read at 450 nm wavelength. A standard curve was always generated with each assay run. Intra-assay coefficients for all the samples ranged from 0.10 to 9.19%. 

Catalase. The catalase activity of serum samples was determined using a Catalase Assay kit (Cayman Chemical, Ann Arbor, MI, USA) following the manufacturer’s instructions and previously described methodologies [29,30,50]. All reagents were equilibrated to room temperature before beginning the assay. Briefly, 20 µL samples, standards, and positive control were diluted in 100 µL assay buffer, which was added to the corresponding wells. Subsequently, 30 µL methanol was poured into each well, and the reaction took place by adding 20 µL hydrogen peroxide. Following a 20- minute incubation on a shaker at room temperature, 30 µL potassium hydroxide was added to terminate the reaction. Subsequently, 30 µL of catalase purpald (chromogen) was incubated with the samples for ten minutes. Finally, 10 µL catalase potassium periodate was incubated with the samples at room temperature for 5 min and the absorbances were read at 540 nm wavelength. A standard curve was always generated with each assay run. Intra-assay coefficients of variation for all the samples ranged from 0.02% to 9.78%.

Superoxide Dismutase (SOD). SOD activity in serum was analyzed using the Superoxide Dismutase Assay Kit (Cayman Chemical, Ann Arbor, MI, USA) following the manufacturer’s instructions and previously described methodologies [29,30,50]. The reagents were equilibrated to room temperature before beginning the assay. Briefly, serum was diluted 1:5 with sample buffer before assaying for SOD activity. Subsequently, 10 µL of the diluted samples, standards, and 200 µL of the diluted Radical Detector was added to the designated wells on the plates. To initiate the enzymatic reaction, 20 µL xanthine oxidase was added to the wells. The samples were incubated at room temperature for 30 min, and the absorbances were read at 440 nm. A standard curve was always generated with each assay run. Intra-assay coefficients of variation for all the samples ranged from 0.06% to 9.57%.

Serum levels of Trolox Equivalent Antioxidant Capacity (TEAC). Total antioxidant capacity within serum samples was analyzed, measuring the levels of TEAC using the OxiSelect^TM^ Trolox Equivalent Antioxidant Capacity TEAC Assay Kit (ABST) (Cell Biolabs, Inc., San Diego, CA, USA) following the manufacturer’s instructions. Briefly, 25 µL of the 20-fold diluted serum samples was added to each well. Subsequently, 150 µL of the diluted 2,2′-azino-bis (3-ethylbenzothiazoline-6-sulfonic acid) (ABTS) reagent was mixed vigorously with the samples to be incubated for five minutes. Finally, the absorbances were read at 405 nm wavelength. A standard curve was always generated with each assay run. Antioxidant activity was determined by comparison with the Trolox standards. Intra-assay coefficients of variation for all the samples ranged from 0.04% to 4.83%.

### 2.8. Statistical Analysis

The normality of all the variables was tested using the Shapiro–Wilk test and histograms. Hepcidin, a key regulator of iron entry into circulation and a reliable indicator of iron status in humans [48], was selected as the target variable to calculate the sample size. To accept an alpha risk of 0.05 and a beta risk of 0.2 (80% power) in a two-sided test, a minimum of 21 patients in the placebo arm and 42 patients in the iron arm were required to detect a minimum difference of 150 ng/mL hepcidin levels. Statistical significance was established at *p* ≤ 0.05. Delta values were also calculated for all the clinical and biological variables. Clinical parameters, iron status, and IL-6 are expressed as mean (standard deviation), while the remainder of the variables are expressed as median and interquartile ranges. The following comparisons in the two study groups were made: (1) at baseline between the two study groups using independent-sample Student’s T-tests, (2) post-treatment between the two study groups using either parametric (independent-sample Student’s T-test) and non-parametric (Mann–Whitney U test) tests as required, (3) delta changes between the two study groups, using either parametric (independent-sample Student’s T-test) and non-parametric (Mann–Whitney U test) tests as required, and (4) within each particular group, post- and pre-treatment comparisons using either parametric (paired T-test) and non-parametric (Wilcoxon signed-rank test) tests as required. Clinical data are shown in two tables, while biological results are illustrated in violin plots: absolute values in A panels and delta change values in B panels. 

Pearson’s correlation coefficients were also determined between serum hepcidin-25 levels and the other study variables, including those of iron metabolism. All the statistical analyses were performed using the software SPSS (Version 23.0, SPSS Inc., Chicago, IL, USA).

## 3. Results

### 3.1. Clinical Characteristics at Baseline

A total of 66 patients participated in the study (*n* = 44 in the iron-treated group). No statistically significant differences were seen in any of the anthropometric, smoking history, or lung function variables at baseline (Table 1). The number of female and male patients did not significantly differ between the two study groups (Table 1). 

### 3.2. Clinical Characteristics Following Iron Replacement

**Figure 2:**
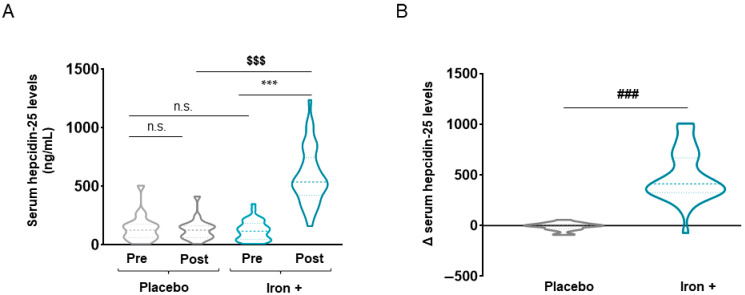
Violin plots of absolute hepcidin-25 levels in serum of all COPD patient groups (**A**) and delta (post-pre) of serum hepcidin-25 levels in placebo and iron-treated groups (**B**). The dashed line represents the median and the dotted lines the interquartile ranges. Statistical significance: n.s., no significance; $$$ *p* < 0.001 between post-iron treatment and post-placebo treatment, *** *p* < 0.001 between post-iron and pre-iron treatment; ### *p* < 0.001 between iron and placebo treatment.

In iron-treated COPD patients, the following parameters were modified after iron replacement compared to baseline: MCV, MCH, serum iron, ferritin, transferrin saturation, transferrin, and soluble transferrin receptor (Table 2). At the post-treatment time-point, the following markers were also modified in the iron-treated patients compared to the placebo group: MCH, serum iron, ferritin, transferrin saturation, and transferrin (Table 2). Moreover, deltas obtained from the difference between post-iron and baseline values were statistically different between iron-treated and placebo groups for the following variables: MCV, MCH, serum iron, ferritin, transferrin saturation, transferrin, and soluble transferrin receptor (Table 2). Interestingly, in the iron-treated patients, absolute serum hepcidin levels were significantly higher after iron replacement than baseline levels (Figure 2A). Furthermore, the delta of hepcidin levels between baseline and post-iron replacement was also significantly greater in the iron-treated than in the placebo COPD patients (Figure 2B). Absolute serum hepcidin post-treatment levels significantly and positively correlated with ferritin serum levels (r = 0.699, *p* = 0.000), while negatively correlated with soluble transferrin receptors and almost significantly with transferrin levels (r = −0.332, *p* = 0.048 and r = −0.273, *p* = 0.08, respectively). Levels of the cytokine IL-6 did not differ between the study groups for any of the time-points (Table 2). 

#### 3.2.1. Oxidative Stress Markers

**Figure 3:**
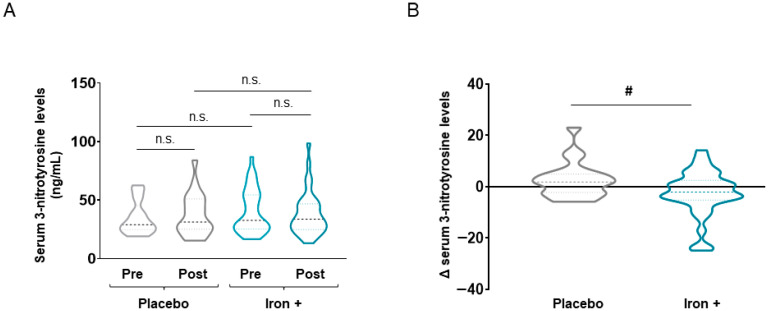
Violin plots of absolute 3-nitrotyrosine levels in serum of all COPD patient groups (**A**) and delta (post-pre) of serum 3-nitrotyrosine levels in placebo and iron-treated groups (**B**). The dashed line represents the median and the dotted lines the interquartile ranges. Statistical significance: n.s., no significance; # *p* < 0.05 between iron and placebo treatment.

**Figure 4:**
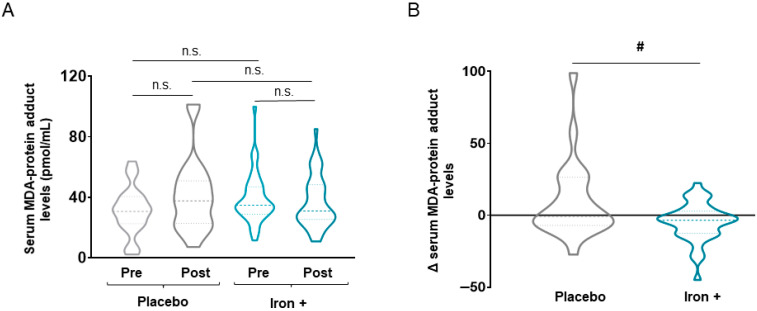
Violin plots of absolute MDA-protein adduct levels in serum of all COPD patient groups (**A**) and delta (post-pre) of serum MDA-protein adduct levels in placebo and iron-treated groups (**B**). The dashed line represents the median and the dotted lines the interquartile ranges. Statistical significance: n.s., no significance; # *p* < 0.05 between iron and placebo treatment. Definition of abbreviations: MDA, malondialdehyde.

**Figure 5:**
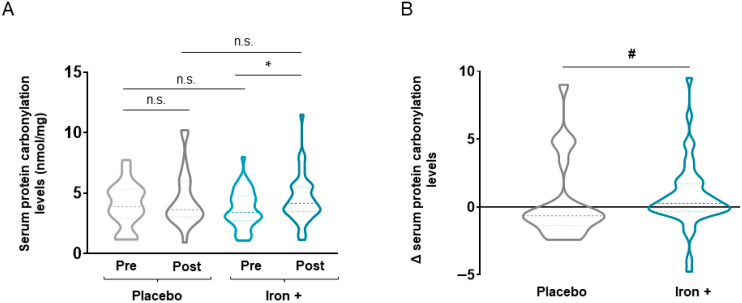
Violin plots of absolute protein carbonylation levels in serum of all COPD patient groups (**A**) and delta (post-pre) of serum protein carbonylation levels in placebo and iron-treated groups (**B**). The dashed line represents the median and the dotted lines the interquartile ranges. Statistical significance: n.s., no significance; * *p* < 0.05 between post-iron and pre-iron treatment; # *p* < 0.05between iron and placebo treatments.

Absolute serum levels of both 3-nitrotyrosine and MDA-protein adducts were not significantly modified either at baseline or following iron replacement in any of the two study groups (Figure 3A and Figure 4A, respectively). Nonetheless, a significant decline in both serum 3-nitrotyrosine and MDA-protein adduct levels were detected in the iron-treated group compared to the placebo when delta values were calculated (Figure 3B and Figure 4B, respectively). Interestingly, in the iron-treated patients, absolute serum levels of reactive carbonyls significantly increased following treatment compared to baseline, while no significant differences were seen in the placebo group of patients (Figure 5A). Furthermore, the delta values were also significantly increased in the iron-treated group compared to the placebo patients (Figure 5B).

#### 3.2.2. Antioxidant Markers

**Figure 6:**
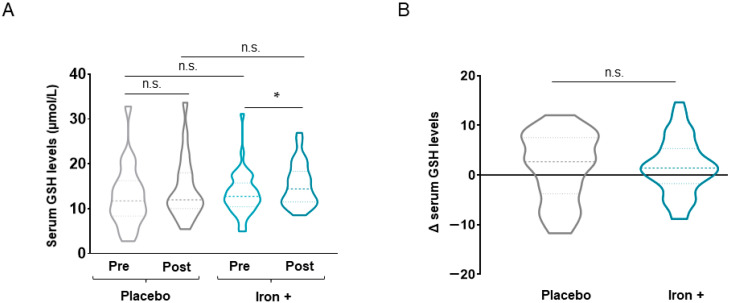
Violin plots of absolute GSH levels in serum of all COPD patient groups (**A**) and delta (post-pre) of serum GSH levels in placebo and iron-treated groups (**B**). The dashed line represents the median and the dotted lines the interquartile ranges. Statistical significance: n.s., no significance; * *p* < 0.05 between post-iron and pre-iron treatment. Definition of abbreviations: GSH, reduced glutathione.

In the iron-treated patients, absolute serum levels of the antioxidant GSH increased following iron replacement compared to baseline, whereas no differences were detected in the placebo patients (Figure 6A). The delta values did not differ between placebo and iron-treated COPD patients (Figure 6B).

**Figure 7:**
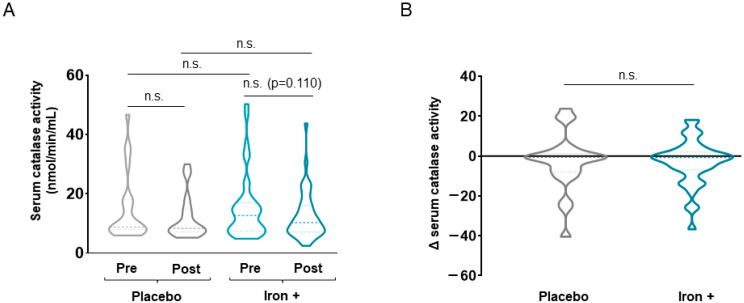
Violin plots of absolute catalase activity in serum of all COPD patient groups (**A**) and delta (post-pre) of serum catalase activity levels in placebo and iron-treated groups (**B**). The dashed line represents the median and the dotted lines the interquartile ranges. Statistical significance: n.s., no significance.

**Figure 8:**
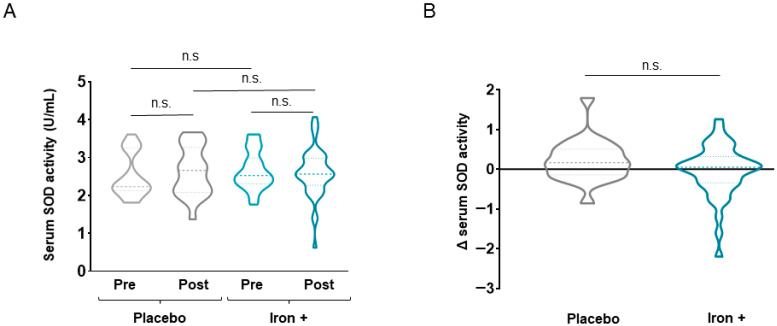
Violin plots of absolute SOD activity in serum of all COPD patient groups (**A**) and delta (post-pre) of serum SOD activity levels in placebo and iron-treated groups (**B**). The dashed line represents the median and the dotted lines the interquartile ranges. Statistical significance: n.s., no significance. Definition of abbreviations: SOD, superoxide dismutase.

**Figure 9:**
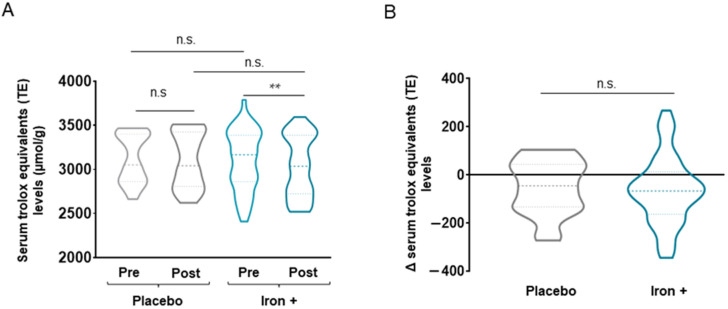
Violin plots of absolute Trolox equivalents levels in serum of all COPD patient groups (**A**) and delta (post-pre) of serum Trolox equivalents levels in placebo and iron-treated groups (**B**). The dashed line represents the median and the dotted lines the interquartile ranges. Statistical significance: n.s., no significance; ** *p* < 0.01 between post-iron and pre-iron treatment.

No statistically significant differences were seen in absolute serum catalase or SOD levels between time-points in the treated group of patients (*p* = 0.110 for catalase levels, Figure 7 and Figure 8, respectively). Delta values did not differ between placebo and treated patients for either catalase or SOD serum levels (Figure 7 and Figure 8). Absolute serum Trolox levels significantly declined following iron replacement in the treated patients compared to baseline, while no differences were seen in the placebo group (Figure 9A). No differences between placebo and iron-treated patients were detected in delta values of Trolox serum levels (Figure 9B).

## 4. Discussion

In the current investigation, the most relevant findings were that iron replacement induced a series of modifications in markers of iron metabolism as well as in red blood cell features. These results evidenced that treatment was effectively administered in the patients. Importantly, a significant decline in the markers of oxidative stress along with an increase in the antioxidant GSH was detected in the patients treated with iron compared to the placebo control group. A discussion of the most relevant results follows.

The clinical characteristics of the patients were similar in both groups. Following iron replacement, markers of iron metabolism significantly improved compared to baseline in the treated patients. Moreover, the delta change of the iron metabolism variables between the iron-treated and the placebo groups of patients also significantly improved in the former patients compared to the latter. These findings are consistent with previous investigations conducted in patients with liver surgery and anemic diseases [28,51,52,53]. Collectively, these results suggest that iron replacement favored the optimization of iron metabolism even in non-anemic patients. Interestingly, no significant differences were seen in IL-6 levels after treatment with iron, findings that had also been previously reported [51].

NAID is very prevalent among patients with COPD [54,55]. NAID can also be underdiagnosed in these patients as a result of an increase in ferritin levels related to inflammatory processes, which would enhance its prevalence even more in actual clinical settings [11,56]. Among the potential therapeutic strategies, intravenous iron therapy was shown to exert beneficial effects in terms of clinical symptoms and quality of life in patients with chronic heart failure with and without anemia [57]. In the current clinical investigation, intravenous iron therapy was well tolerated in the treated patients, and their clinical symptoms also improved after the trial [35].

Significant associations among indicators of iron metabolism status were detected in the study. As such, hepcidin significantly correlated with ferritin and the soluble transferrin receptor in patients treated with iron. These findings are similar to those encountered previously [58,59,60], in which hepcidin levels also correlated with several iron parameters. Collectively, these results point towards the potential value of hepcidin as an indicator of iron status in patients with chronic respiratory diseases. Future studies should target hepcidin as a biomarker of iron metabolism and prognosis in chronic disease.

In the present study, in the iron-treated group, serum oxidative stress levels significantly declined as measured by 3-nitrotyrosine and MDA-protein adduct markers. Furthermore, the serum levels of the powerful antioxidant GSH increased in the iron-treated group. Similar results were found in iron-deficient anemic patients, in whom MDA-protein adducts also decreased following iron replacement [37]. Collectively, these findings suggest that iron replacement may have contributed to lowering the levels of oxidative stress in COPD patients in as low as four weeks (duration of the study protocol). As redox signaling and oxidative stress are related to iron availability [61], restoration of the iron deposits following iron replacement may have contributed to a reduction in serum oxidative stress levels in COPD patients [62]. The results obtained in the study are also consistent with those previously reported [36], in which several oxidative stress markers were reduced in response to iron replacement.

A rise in serum reactive carbonyl levels, however, was detected in the iron-treated group following intravenous iron administration for four weeks. These findings are consistent with previously reported studies in which protein carbonylation levels also increased following iron replacement in patients with renal failure [63] and anemic patients [64]. The fact that more elemental free iron becomes available in response to iron treatment may account for the rise in reactive carbonyls detected in the iron-treated patients. Further research should focus on the elucidation of the mechanisms that underlie increased reactive carbonyls in the serum compartment of patients receiving iron therapy. 

### Study Limitations

One of the limitations is related to the fact that the clinical trial was single-blind and was only conducted in one center. Nonetheless, evidence has been obtained from a randomized clinical trial in which severe COPD patients with NAID were carefully selected. Another limitation may be related to the fact almost half of the patients in each group were active smokers. Nonetheless, as smoking history did not significantly differ between the two study groups, this issue may not have had any substantial implications in the study results. Moreover, the target population represents a realistic cohort of COPD patients. Interestingly, the proportions of male and female patients were similar in both study groups. Thus, gender differences probably did not play a significant role in the study. In summary, the results reported herein may serve as the basis for the design of future research at a multicentric level.

## 5. Conclusions

This randomized clinical trial evidenced that iron replacement elicited a decline in serum oxidative stress markers along with an improvement in serum GSH levels in patients with stable severe COPD. Hepcidin may be a surrogate biomarker of iron status and metabolism in patients with chronic respiratory diseases. These findings have potential clinical implications in the management of patients with severe COPD, as NAID is a prevalent systemic manifestation in these patients.

## Figures and Tables

**Table 1:** Clinical characteristics of the study patients at baseline.

	Placebo (*N* = 22)	Iron (*N* = 44)
Anthropometry		
Age, years	69.2 (5.4)	66.8 (7.3)
Males/females	16/6	26/18
BMI, kg/m^2^	25.1 (2.9)	24.4 (3.4)
FFMI, kg/m^2^	16.14 (1.8)	15.9 (2.1)
Smoking status		
Ex-smoker, N (%)	13 (59.1)	20 (45.5)
Active, N (%)	9 (40.9)	24 (54.5)
Packs-year	56.3 (36.0)	51.5 (25.8)
Pulmonary Function		
FEV_1_, L	1.3 (0.3)	1.4 (0.6)
FEV_1_, % predicted	47.0 (10.6)	51.6 (13.5)
FVC, L	3.0 (0.7)	2.8 (0.8)
FVC, %	84.5 (13.1)	80.7 (12.6)
FEV_1_/FVC, %	43.5 (9.1)	48.7 (10.4)
RV, %	175.9 (40.6)	176.3 (44.4)
RV/TLC, %	59.3 (7.1)	60.0 (12.8)
TLC, %	108.5 (15.1)	110.4 (16.1)
DL_CO_, %	53.0 (16.7)	52.3 (12.6)
KCO, %	57.1 (16.2)	55.0 (12.4)
Arterial blood gas		
PaO_2_, kPa	10.4 (0.9)	10.2 (1.1)
PaCO_2_, kPa	5.1 (0.6)	5.1 (0.5)

Data are presented as mean (SD). Abbreviations: BMI, body mass index; FFMI, fat-free mass index; N, number of patients; FEV1, forced expiratory volume in 1 s; FVC, forced vital capacity; RV, residual volume; TLC, total lung capacity; DLCO, carbon monoxide diffusion capacity corrected for hemoglobin concentration; KCO, Krough transfer factor; PaO2, oxygen partial pressure; PaCO2, carbon dioxide partial pressure.

**Table 2:** Iron metabolism and inflammatory parameters of the study patients at baseline and after treatment.

	Placebo (*N* = 22)			Iron (*N* = 44)		
	Pre	Post	Δ	Pre	Post	Δ
Iron status						
Hemoglobin, g/dL	14.9 (1.2)	15.0 (1.2)	+0.1 (0.7)	14.5 (1.2)	15.7 (4.8)	+1.3 (4.8)
Hematocrit, %	45.0 (3.5)	45.5 (3.6)	+0.5 (2.8)	43.6 (3.7)	45.1 (4.0) ***	+1.6 (2.3)
MCV, fl	90.3 (4.7)	89.8 (5.7)	−0.5 (2.2)	90.6 (3.7)	91.9 (3.8) ***	+1.4 (1.8) ^###^
MCH, pg	29.8 (2.1)	29.6 (2.3)	−0.3 (0.7)	30.1 (1.7)	30.6 (1.5) ***^,$^	+0.5 (0.8) ^###^
MCHC, g/dL	33.0 (1.1)	32.9 (1.2)	−0.2 (1.1)	33.2 (1.0)	33.3 (1.1)	+0.1 (0.8)
Serum iron, µg/dL	83.8 (36.4)	89.3 (26.0)	+5.4 (37.1)	82.5 (32.5)	108.7 (31.2) ***^,$^	+26.2 (36.4) ^#^
Ferritin, ng/mL	69.0 (42.4)	64.3 (41.6)	−4.6 (18.4)	61.9 (36.2)	342.2 (176.0) ***^,$$$^	+280.3 (178.9) ^###^
Transferrin saturation, %	22.6 (10.1)	23.2 (7.4)	+0.5 (9.6)	22.5 (11.1)	34.8 (11.1) ***^,$$$^	+12.2 (11.0) ^###^
Transferrin, g/dL	271.3 (36.9)	277.0 (29.2)	+8.6 (23.7)	277.0 (41.9)	227.5 (33.5) ***^,$$$^	−49.5 (38.0) ^###^
Soluble transferrin receptor, mg/L	3.2 (1.6)	3.3 (1.8)	+0.3 (0.8)	3.3 (2.1)	2.7 (1.4) **	−0.6 (1.1) ^###^
Inflammatory marker						
IL-6, pg/mL	14.0 (17.6)	12.5 (17.7)	−0.7 (13.2)	12.9 (19.9)	11.4 (9.8)	1.8 (8.9)

Data are presented as mean (SD). Abbreviations: MCV, mean corpuscular (erythrocyte) volume; MCH, mean corpuscular hemoglobin; MCHC, mean corpuscular hemoglobin concentration; Statistical significance: ** *p* ≤ 0.01, *** *p* ≤ 0.001 between post-iron treatment and pre-iron treatment; ^$^ *p* ≤ 0.05, ^$$$^ *p* ≤ 0.001 between post-iron treatment and post-placebo treatment; ^#^ *p* ≤ 0.05, ^###^ *p* ≤ 0.001 between iron and placebo groups. Systemic redox balance in the study patients.

## Data Availability

The datasets are available from the corresponding authors upon reasonable request.
